# Silicate Microfiber Scaffolds Support the Formation and Expansion of the Cortical Neuronal Layer of Cerebral Organoids With a Sheet-Like Configuration

**DOI:** 10.1093/stcltm/szad066

**Published:** 2023-10-16

**Authors:** Eisaku Terada, Yohei Bamba, Masatoshi Takagaki, Shuhei Kawabata, Tetsuro Tachi, Hajime Nakamura, Takeo Nishida, Haruhiko Kishima

**Affiliations:** Department of Neurosurgery, Osaka University Graduate School of Medicine, Osaka, Japan; Department of Neurosurgery, Osaka University Graduate School of Medicine, Osaka, Japan; Department of Neurosurgery, Osaka University Graduate School of Medicine, Osaka, Japan; Department of Neurosurgery, Osaka University Graduate School of Medicine, Osaka, Japan; Department of Neurosurgery, Osaka University Graduate School of Medicine, Osaka, Japan; Department of Neurosurgery, Osaka University Graduate School of Medicine, Osaka, Japan; Department of Neurosurgery, Osaka University Graduate School of Medicine, Osaka, Japan; Department of Neurosurgery, Osaka University Graduate School of Medicine, Osaka, Japan

**Keywords:** induced pluripotent stem cells, organoids, neurogenesis, neurons, tissue scaffolds

## Abstract

Cerebral organoids (COs) are derived from human-induced pluripotent stem cells in vitro and mimic the features of the human fetal brain. The development of COs is largely dependent on “self-organization” mechanisms, in which differentiating cells committed to cortical cells autonomously organize into the cerebral cortex-like tissue. However, extrinsic manipulation of their morphology, including size and thickness, remains challenging. In this study, we discovered that silicate microfiber scaffolds could support the formation of cortical neuronal layers and successfully generated cortical neuronal layers, which are 9 times thicker than conventional COs, in 70 days. These cortical neurons in the silicate microfiber layer were differentiated in a fetal brain-like lamination pattern. While these cellular characteristics such as cortical neurons and neural stem/progenitor cells were like those of conventional COs, the cortical neuronal layers were greatly thickened in sheet-like configuration. Moreover, the cortical neurons in the scaffolds showed spontaneous electrical activity. We concluded that silicate microfiber scaffolds support the formation of the cortical neuronal layers of COs without disturbing self-organization-driven corticogenesis. The extrinsic manipulation of the formation of the cortical neuronal layers of COs may be useful for the research of developmental mechanisms or pathogenesis of the human cerebral cortex, particularly for the development of regenerative therapy and bioengineering.

## Graphical Abstract

**Graphical Abstract:**
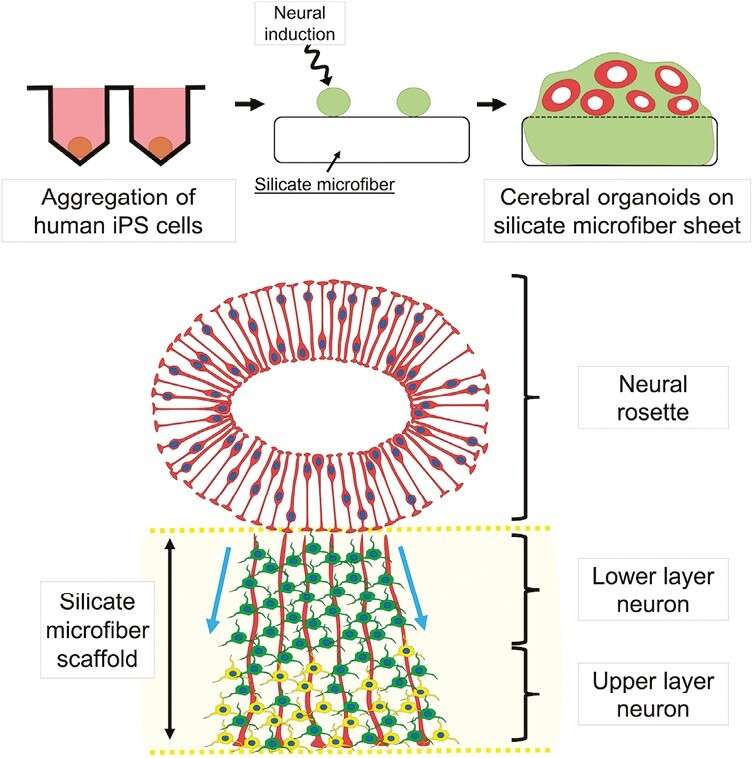
Cerebral organoids derived from induced pluripotent stem cells are cultured on silicate microfiber scaffolds. Cells produced by cerebral organoids migrate into the silicate microfiber scaffolds and form thick cortical neuronal layers. Cell diversity, lamination patterns, and neuronal layer size are reminiscent of the human fetal cortex.

**Significance Statement**

The cortical neuronal layer of cerebral organoids is relatively thinner and smaller than that of the fetal human brain, which can be a barrier to its biological or clinical application. In this study, we successfully established a method to support the formation of cortical neuronal layers in cerebral organoids using silicate microfiber scaffolds. Using this method, thick cortical neuronal layers with a sheet-like configuration could be generated from the cerebral organoids derived from human-induced pluripotent stem cells.

## Introduction

Recent advances in stem cell technology have enabled the generation of cerebral cortex-like tissues in vitro via an autonomous process called “self-organization” from human-induced pluripotent stem cells (hiPSCs). These cortical tissues are called cerebral organoids (COs). They contain various cell types generally seen during fetal corticogenesis, which play important roles in the formation of gyrated and layered human cerebral cortex.^1-3^ Transcriptome analyses showed that they are similar to developmental trajectories.^4-6^ COs have been used in human brain research, including in vitro disease modeling,^3,7-10^ evolutionary biology,^11,12^ drug screening,^13^ and regenerative therapy for stroke and traumatic brain injury.^14,15^ Moreover, they have become indispensable tools in the research of the human central nervous system.

The enlargement of COs was achieved using a laminin-based extracellular matrix^16^ and poly lactic-co-glycolic acid scaffold,^17^ which successfully promoted the growth of the neuroepithelium. However, the size and configuration of COs are still not comparable to those of the human adult brain. Each CO had a thinner cortical layer than that of the human cerebral cortex,^18,19^ which fuse with one another in a disorderly manner. This disordered configuration is an obstacle in conforming COs to regenerative therapy in the cerebral cortex or in the research of human cortical development or diseases at later developmental stages.

In this study, we hypothesized that an appropriate microenvironment and mechanical support by robust scaffolds could promote and regulate the formation of the cortical neuronal layer of COs. To overcome the limitations of previous methods, which are mainly based on self-organization-driven corticogenesis in biodegradable extracellular matrices only, we aimed to enable the expansion of the cortical layer in a sheet-like configuration using silicate microfiber (SiF) scaffolds. The use of SiF scaffolds for growing COs may be a promising approach in the research on human corticogenesis, pharmaceutical applications, and regenerative medicine for diseases in the cerebral cortex.

## Materials and Methods

### Maintenance of hiPSCs

All cell cultures were maintained in a 5% CO_2_ incubator at 37°C. Human iPSCs (201B7, 409B2, and 1201C1) were provided by RIKEN BRC (Tsukuba, Japan) through the National Bio Resource Project of the Ministry of Education, Culture, Sports, Science and Technology/Agency for Medical Research and Development, Japan.^20^ The iPSCs were maintained on the feeder layer of mitotically inactivated SL10 (Reprocell, Kyoto, Japan) and cultured in primate embryonic stem cell medium (Reprocell) with 5 ng/mL basic fibroblast growth factor (Reprocell), according to the manufacturer’s protocol. Passages were performed every 5 days, and cells at passage 4 to 30 were used in subsequent experiments.

### Generation of Silicate Microfiber Non-Woven Fabric (SiF) Sheets

All silicate microfiber non-woven fabric sheets were prepared using a modification of previously reported method.^21^ Specifically, silica sol was created by heating a mixture of tetraethoxysilane, water, ethanol, and hydrogen chloride (molar ratio = 1:2:5:0.003). Subsequently, it was subjected to electrospinning and then sintered at 800°C to yield SiF sheets with an average fiber diameter of 1 µm. All SiF sheets were fabricated by the manufacturer (Japan Vilene, Tokyo, Japan). Some experiments were performed using a thicker variant of SiF sheet, fabricated by increasing the SiF content to 28 g/m^2^, while the SiF content in the standard product was 8 g/m^2^.

### Generation of COs on SiF Sheets

**Figure 1.**
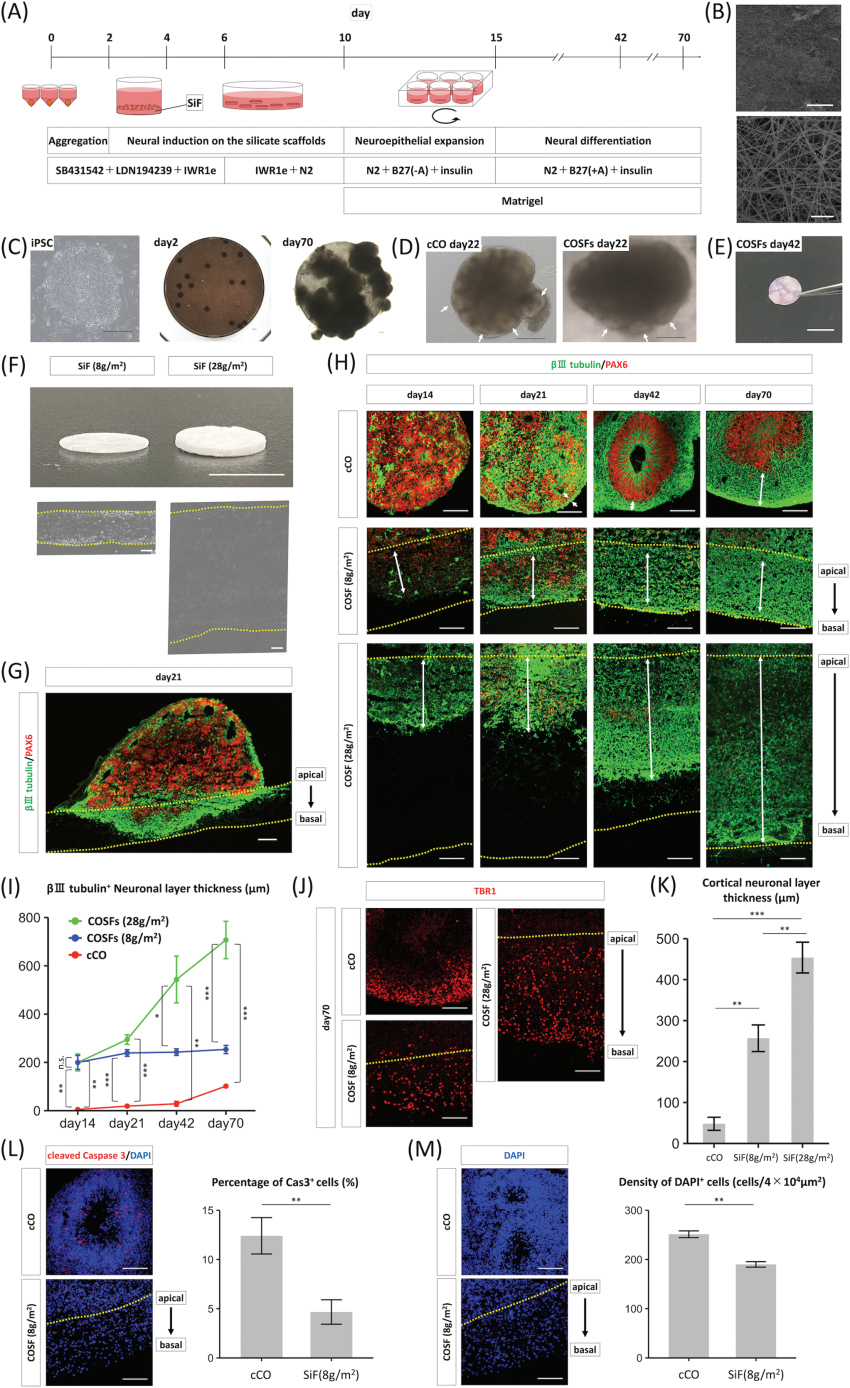
Derivation of cerebral organoids on silicate microfiber sheets (COSFs). (**A**) Procedure for the derivation of cerebral organoids (COs) on silicate microfiber (SiF) non-woven fabric. (**B**) Electron microscopy image of the SiF sheet. Scale bar = above 500 µm, below 10 µm. (**C**) Phase-contrast images of undifferentiated-induced pluripotent stem cells and COSFs on days 2 and 70. Scale bar = 300 µm. (**D**) Phase-contrast images of COs and COSFs on day 22. COs and COSFs have many neuroepithelia (white arrows). Scale bar = 300 µm. (**E**) Picture of whole COSF. COSFs could be easily grasped using forceps without collapse. Scale bar = 5 mm. (**F**) Photographs and phase-contrast images of standard- and thick-type SiFs. The thickness of the standard-type was 262.9 ± 23.5 µm, while that of the thick-type was 822.9 ± 39.9 µm. *n* = 3 points each from 8 individual SiF sheets. Scale bar = above 5 mm, below 100 µm. (**G**) Immunohistochemistry of COSFs (standard-type) and COs for βIII tubulin and PAX6 at 3 weeks. βIII tubulin-positive neuronal cells reached the basal side of the SiF sheet. (**H**) Immunohistochemistry of COSFs and COs for βIII tubulin and PAX6 during 10 weeks. The dotted line indicates the apical border of an SiF sheet. Scale bar = 100 µm. (**I**) Expansion of the βⅢ tubulin-positive neuronal cell layer in COSFs and cCOs during long-term culture. *n* = 3 individual COSFs and 3 individual cCOs. Results are presented as mean ± SEM. **P < 001; ***P < .001, the comparisons of each day were performed using Tukey-Kramer tests. (**J**) Distribution of the cortical neurons (TBR1-positive nuclei) in the COSFs and cCOs. The dotted line indicates the apical border of an SiF sheet. Scale bar = 100 µm. (**K**) Comparison of TBR1-positive cortical neuronal layer thickness among COs and COSFs. In 70 days, the cortical thickness was 453.8 ± 37.6 μm (COSFs with thick-type SiF sheets), 256.9 ± 32.6 μm (COSFs with standard-type SiF sheets), and 48.1 ± 15.9 μm (cCOs). *n* = 3 individual COs and 3 points from three individual COSFs. Results are presented as mean ± SEM. ***P < .001, Tukey-Kramer test. (L) Comparison of the viability rates between cCOs and COSFs. The dotted line indicates the apical border of an SiF sheet. *n* = 6 individual cCO and COSFs values. Results are presented as mean ± SEM. **P < .01, Student’s *t* test. (**M**) Comparison of the cell densities of cCOs and COSFs. The dotted line indicates the apical border of an SiF sheet. *n* = 3 individual cCO and COSFs values. Results are presented as mean ± SEM. **P < .01, Student’s *t* test.

On day 0, iPSC colonies were separated into single cells by enzymatic dissociation with 0.25% trypsin-EDTA (Thermo Fisher Scientific, Waltham, MA, USA) after treatment with 10 μM Y27632 (Nacalai Tesque, Kyoto, Japan) for 30 minutes. To form the aggregation of iPSCs, 12 000 cells were plated in each well of an ultra-low attachment 96-well plate with V-bottomed conical wells (Sumitomo Bakelite, Tokyo, Japan) in 100 μL aggregation medium containing Glasgow’s Minimum Essential Medium (Sigma-Aldrich, St.Louis, MO, USA), 20% [v/v] KnockOut serum replacement (Thermo Fisher Scientific), 1% [v/v] Minimum Essential Medium Non-essential Amino Acid Solution (MEM-NEAA) (Thermo Fisher Scientific), 1 mM sodium pyruvate (Thermo Fisher Scientific), 100 U/mL penicillin, 100 μg/mL streptomycin, 250 ng/mL amphotericin B (Thermo Fisher Scientific), 1 μM 2-mercaptoethanol (Wako, Osaka, Japan), 3 μM IWR1-endo (Wako), 100 nM LDN-193189 (Stemgent, MA, USA), 5 μM SB431542 (Stemgent), and 30 μM Y27632 (Nacalai Tesque). On day 2, 16 aggregates were transferred along with the medium using a 1000μL micropipette onto 2 types of SiF sheets in an ultra-low attachment 96-well plate (Corning, NY, USA), with the aggregation medium changed twice a day. On day 4, COs on silicate microfiber sheets (COSFs) were transferred to an ultra-low attachment 60 mm dish (Corning). On day 6, the aggregation medium was switched to an induction medium containing Dulbecco’s Modified Eagle’s Medium (DMEM)/F12/GlutaMAX (Thermo Fisher Scientific), 1% [v/v] N2 supplement (Thermo Fisher Scientific), 1% [v/v] MEM-NEAA (Thermo Fisher Scientific), 100 U/mL penicillin, 100 μg/mL streptomycin, 250 ng/mL amphotericin B, 3 μM IWR1-endo, and 1 μg/mL heparin (Sigma-Aldrich). On day 10, COSFs were embedded in Matrigel (Corning,) and transferred to ultra-low attachment 6-well plates (Corning) in an expansion medium containing a 1:1 mixture of DMEM/F12 (Sigma-Aldrich) and Neurobasal medium (Thermo Fisher Scientific), 0.5% [v/v] N2 supplementation, 0.025% [w/v] insulin (Sigma-Aldrich), 1% [v/v] GlutaMAX (Thermo Fisher Scientific), 1% [v/v] MEM-NEAA, 100 U/mL penicillin, 100 μg/mL streptomycin, 250 ng/mL amphotericin B, 1 μM 2-mercaptoethanol (Wako), and 1% [v/v] B27 supplement minus vitamin A (Thermo Fisher Scientific). Culture dishes were placed on a rotary shaker (Taitec, Saitama, Japan) at 40*g* and placed in 5% CO_2_ incubators at 37°C. On day 15, the expansion medium was switched to a differentiation medium containing a 1:1 mixture of DMEM/F12 and Neurobasal medium, 5% [v/v] N2 supplementation, 0.025% [w/v] insulin (Sigma-Aldrich), 1% [v/v] GlutaMAX, 1% [v/v] MEM-NEAA, 100 U/mL penicillin, 100 μg/mL streptomycin, 250 ng/mL amphotericin B (Thermo Fisher Scientific), 1 μM 2-mercaptoethanol (Wako), and 1% [v/v] B27 supplement (Thermo Fisher Scientific). After day 15, the medium was changed twice a week. These procedures are summarized (Fig. 1A) and were modified according to previous reports.^2,16^ For the control experiments, spherical COs were generated using the same protocol without SiF sheet (henceforth referred to as conventional COs [cCOs]).

### Quantitative Reverse Transcription-Polymerase Chain Reaction

Total RNA was extracted using the RNeasy Mini Kit (Qiagen, Venlo, Netherlands). All the COSFs, including SiF sheets, were lysed by pipetting according to the previous report.^22^ Contaminated DNA was removed using the RNase-free DNase set (Qiagen). cDNA was synthesized using the PrimeScript RT reagent kit (Takara Bio, Shiga, Japan). Quantitative PCR was performed using the Power SYBR Green PCR Master Mix (Thermo Fisher Scientific) and Quant Studio 7 (Thermo Fisher Scientific). Cycles were run at 95°C denaturation for 15 seconds and 60°C annealing for 45 seconds at a total of 40 cycles. The primer pairs used have been previously reported (Supplementary Table S1). The expression of each gene was normalized by that of glyceraldehyde-3-phosphate dehydrogenase, and the relative expression was determined using the ^ΔΔ^CT method as previously described.^23,24^

### Immunohistochemistry

cCOs and COSFs were fixed in 4% paraformaldehyde and cryoprotected in 30% [w/v] sucrose solution at 4°C. They were embedded and frozen at −80°C in O.C.T. compound (Sakura Finetek, Tokyo, Japan) and cryosectioned to 10-12 μm. The tissue sections were permeabilized in 0.3% Triton-X/phosphate-buffered saline (Nacalai Tesque) and blocked with 10% goat serum (Sigma-Aldrich). The primary and secondary antibodies and their dilutions are listed in Supplementary Table S2. Nuclear staining was performed with 4ʹ,6-diamidino-2-phenylindole dihydrochloride (DAPI) (Nacalai Tesque). The samples were examined using the fluorescence microscope BZ-X700 or BZ-X800 (Keyence, Osaka, Japan) and the confocal laser-scanning microscope LSM710 (Carl Zeiss, Oberkochen, Germany). The presence of the SiF layer in each sample was confirmed using phase-contrast microscopy.

### Immunohistochemical Analysis

The positions of the nuclear markers of cortical neurons (SATB2, TBR1, and CTIP2) in the COSFs were measured based on the distance from the apical (lower) surfaces of the SiF sheet (Fig. 1J). The thickness of TBR1-positive layers was measured as previously described.^25^ cCOs and COSFs at day 70 were immunostained for cleaved-caspase 3, and the positively stained cells were quantified to detect apoptotic cells. Cell counts and measurements were performed using Fiji software (ImageJ; v 1.53a).

### Time-Lapse Imaging for Neurite Extension From COSFs

The COSFs were placed on a Matrigel-coated glass bottom dish (Matsunami Glass, Osaka, Japan). On the next day, they were examined using a microscope, IX83 (Olympus, Tokyo, Japan), equipped with a temperature (37°C) and CO_2_ (5%) control. Time-lapse phase-contrast images were acquired at 5-minute intervals for 4 days. The growth speed of axons was measured in 20 axons at 3 hours. After examination, the samples were fixed and stained as described earlier.

### Multiphoton Microscopy and Calcium Imaging

COSFs at day 70 were stained with 0.25 µM NeuO (STEMCELL Technologies, Vancouver, Canada) and 2 µg/mL DAPI in the expansion medium. After a 1 hour incubation, the medium was switched to an expansion medium without NeuO and DAPI. After 2 hours of incubation, the cells were examined using a multiphoton microscope, A1 MP+ (Nikon, Tokyo, Japan), equipped with a temperature (37°C) and CO_2_ (5%) control. The area within 60 µm in depth from the basal (upper) side was observed. For calcium imaging, live imaging was performed using the multiphoton microscope, A1 MP+. For calcium dye loading, COSFs on days 70-75 and 100-105 were incubated with 1 µM Fluo4-AM solution (Dojindo Laboratories, Kumamoto, Japan) for 60 minutes at 37°C. Excess dye was removed by washing with the expansion medium. Imaging was performed using frames taken every 2 seconds for 10-20 minutes. Images were obtained from the basal side to a depth of 30 µm. Glutamate (Wako) and tetrodotoxin (Wako) were added during imaging at final concentrations of 100 and 1 µM, respectively. Data analysis of calcium imaging was performed using ImageJ (Fiji). Regions of interest were selected manually, and the mean fluorescence (F) was calculated for each frame. The fluorescence changes over time were calculated as follows: ΔF/F = (F − F_basal_)/F_background_, where F_basal_ is the lowest mean fluorescence value across imaging, and F_background_ is the mean fluorescence across all frames.

### Statistical Analysis

Data are presented as mean ± SEM in the figures. Statistical tests were performed using JMP Pro 15 software (SAS Institute Inc., Cary, NC, USA). Statistical significance was tested using Student’s *t*-test for 2-group comparisons. One-way analysis of variance with multiple comparisons using the Tukey-Kramer test was performed to determine whether 3 or more datasets were significantly different. Pearson’s correlation coefficient was calculated to assess the strength of the correlation between the 2 continuous variables of calcium surges. Statistical significance was set at *P* < .05. Signifiers used were as follows: *P < .05, **P < .01, ***P < .001.

## Results

### Generation of COSFs From hiPSCs

Our protocol for generating COSFs involved 3 steps: neural induction, neuroepithelial expansion, and neural differentiation (Fig. 1A). SiF sheets composed of electrospun silicate microfibers have high porosity which permit cell-cell interactions (Fig. 1B). Silicate microfiber is chemically stable and compatible for long-term culture.^26^ COs gradually grew on the SiF sheets (Fig. 1C). The formation of several neural rosettes in each aggregate was observed at approximately 10 days, and the well-elongated neuroepithelium could be observed in COSFs in 3 weeks (Fig. 1D). The COSFs could keep their sheet-like configuration and be grasped using forceps without collapse (Fig. 1E), despite culturing for 70 days owing to the mechanical robustness of SiF sheets.

### SiF Scaffolds Support the Expansion of the Neuronal Cell Layer of COs

COSFs with 2 types of SiF scaffolds, standard and thick type, were generated to estimate the effect of SiF scaffolds on the growth of COs. Thick-type SiF sheets were composed of silicate microfibers with a density of 28 g/m^2^, while standard-type SiF sheets were composed of silicate microfibers with a density of 8 g/m^2^. The sections of both types were made and measured, and the thickness of the thick-type SiF sheets was 3.13 ± 0.15 times that of the standard-type SiF sheets (Fig. 1F).

COSFs were compared with COs that were generated without SiF scaffolds (cCOs). The generation and characterization of cCOs are shown in Supplementary Fig. S1A, S1B, suggesting that cCOs had characteristics similar with that of previously and generally reported COs.^1-3,5^ The down regulation of OCT4 and increased expression of both NS/PCs marker and cortical neuron markers were confirmed (Supplementary Fig. S1C--S1E) by quantitative reverse transcription-polymerase chain reaction (RT-PCR) and immunohistochemistry. For the alignment of the cortical neuron, SATB2-positive upper layer neurons and CTIP2 (or TBR1)-positive deep layer cortical neurons tended to be in the basal or apical sides, respectively (Supplementary Fig. S1F), as reported previously.^2^

Immunohistochemistry of βⅢ tubulin (neuronal marker) and PAX6 (cortical progenitor cell marker) were performed in COSFs and cCOs, in which abundant PAX6-positive neural progenitor cells were differentiated after 14 days. PAX6-positive cortical progenitor cells and βⅢ tubulin-positive neurons appeared and began to form the neuronal cell layer in the SiF sheets of COSFs. The neuronal layer reached the basal side of the standard-type SiF sheets (Fig. 1G, 1H) in 21 days. The βⅢ tubulin-positive neuronal cells reached the basal surface of the thick-type SiF sheets in 70 days (Fig. 1H).

Temporal changes in the thickness of the βⅢ tubulin-positive neuronal cell layer were compared and quantified in both COSFs and COs (Fig. 1I). From the early-stage of COSFs, the neuronal cell layers were highly developed compared with those of cCOs. While the thickness of the neuronal cell layer in the standard-type SiF sheet started leveling off in the first 21 days that in the thick-type SiF sheet continued to increase in 70 days. The TBR1-positive cortical cell layer thickness of COSFs on day 70 was also compared to quantify the exact distribution of cortical neuronal cells (Fig. 1J). TBR1 is an early cortical neuronal marker, which is expressed in the cortical neuron in apically biased locations^2^ but distributed throughout the cortical neuronal layer of COs (Supplementary Fig. S1F). The thickness of the cortical neuronal cell layer in standard-type SiF sheets was 5.34 ± 0.68 times thicker than that of cCOs, suggesting that SiF sheets greatly support the formation of the cortical cell layer of COs. In thick-type SiF sheets, the thickness of the cortical neuronal layer added up to 9.43 ± 0.78 times thicker than that of cCOs (Fig. 1K), which would be equivalent to the thickness of the cortical plate in a developing human neocortex during the early second trimester stage.^27^ In addition, similar results were observed in the 409B7 line (Supplementary Fig. 2A).

A significant difference in cell density between the standard- and thick-type SiF sheets was not observed (cell density: 190 ± 5.6 cells/4 × 10^4^ µm^2^ [standard type] and 188 ± 4.4 cells/4 × 10^4^ µm^2^ [thick-type], *n* = 3 individual COSFs, *P* = .759, Student’s *t*-test), indicating that more neuronal cells were accumulated in thick-type sheets. Thus, we concluded that the cortical neuronal layer could be easily expanded using SiF scaffolds. Cytotoxicity was assessed by detecting cleaved Caspase 3 apoptotic cells because thickened neuronal layers may deteriorate the cellular microenvironment by hindering the diffusion of oxygen and nutrition. The number of cleaved Caspase 3-positive cells in COSFs was remarkably decreased compared to that in cCOs (Fig. 1L, Supplementary Fig. S2B), and the cell density inside COSFs was decreased compared to that in COs (Fig. 1M). This result suggested that the porous structure of SiF scaffolds provided intercellular spaces to cortical neurons, leading to enhanced cell viability. For further experiments, we used COSFs with standard-type SiF sheets, because thick-type SiF sheets were relatively fragile and not suitable for large-scale production.

To validate that the interaction of various scaffolds with cerebral organoids, we replicated the experiments using poly(lactide-co-glycolide) copolymer (PLGA) scaffolds (Ethicon, Somerville, NJ, USA) and hydroxyapatite (HA)/PLGA scaffolds (ORTHOREBIRTH CO. LTD., Kanagawa, Japan). During long-term incubation, the PLGA mesh dissolved (Supplementary Fig. S3A). Conversely, with HA/PLGA scaffolds, the organoids predominantly adhered to the surfaces of the HA/PLGA fibers, while the PAX6-positive neuroepithelium infiltrated the gaps between the thicker HA/PLGA fibers, leading to an inability to form distinct neuronal cell layers within the scaffolds (Supplementary Fig. S3B). Based on these observations, we speculated that the ability to balance fine gaps and structural stability is an important factor in conferring the distinctive effects of SiF sheets.

### Characterization of COSFs With Neural Lineage Markers

**Figure 2.**
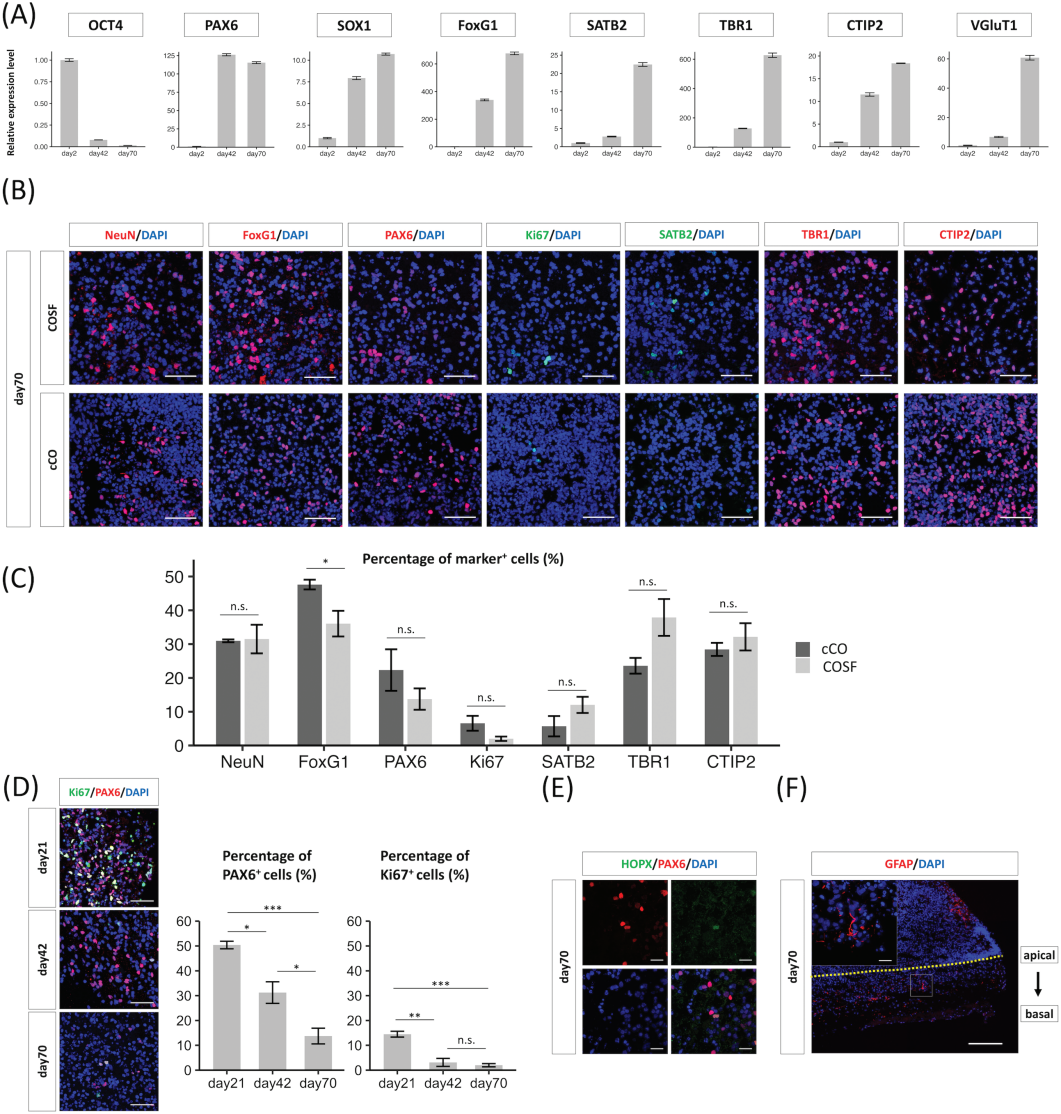
Cortical differentiation and cell diversity of cerebral organoids on silicate microfiber sheets (COSFs). (**A**) Quantitative reverse transcription-polymerase chain reaction analysis for the expression of the marker genes for neural lineage and undifferentiated stem cells in induced pluripotent stem cells (iPSCs) in days 2, 42, and 70 COSFs. Relative expression levels normalized to the expression levels in day 2 are shown as fold change ± SEM, *n* = 3 technical replicates. (**B**, **C**) Immunohistochemistry and quantification of NeuN-, FoxG1-, PAX6-, Ki67-, SATB2-, TBR1-, and CTIP2-positive cells in the SiF layer of COSFs and cCOs at day 70. Scale bar = 50 µm. *n* = 3 individual COSFs and cCOs on day 70. Results are presented as the mean ± SEM. *P < .05, Student’s *t* test. D. Percentage of PAX6^+^ and Ki67^+^ cells normalized with 6-diamidino-2-phenylindole dihydrochloride (DAPI) in the silicate microfiber (SiF) layer on days 21, 42, and 70. *n* = 3 individual COSFs on days 21, 42, and 70. Results are presented as mean ± SEM. *P < .05, **P < .01, ***P < .001, Tukey-Kramer test. (**E**) Immunohistochemistry for PAX6 and HOPX revealed the presence of outer radial glial-like cells in the sheet. Scale bar = 20 µm. HOPX staining accounted for 9.5 ± 4.4% of PAX6-positive cells (*n* = 54 PAX6^+^ cells from 3 individual COSFs). (**F**) Immunohistochemistry for glial fibrillary acidic protein and DAPI revealed that there were only a few astrocytes inside the SiF sheet on day 70. Scale bar = large 200 µm, small 20 µm.

The cellular identity of COSFs was examined using neural lineage markers. Quantitative RT-PCR analysis of COSFs on days 2, 42, and 70 after the initiation of differentiation was performed (Fig. 2A). The expression of the pluripotent stem cell marker, OCT4, was downregulated during the course of COSF generation, whereas the neural stem/progenitor cell (NS/PC) markers, PAX6 and SOX1, were highly expressed at days 42 and 70, indicating that differentiation from iPSC to NS/PCs proceeded during first 6 weeks. In addition, the expression of the forebrain marker, FoxG1, was upregulated over time, indicating that COSFs were regionally patterned to the telencephalon. The expressions of the deep layer (early born) cortical neuronal markers, TBR1 and CTIP2, and upper layer (late-born) cortical neuronal marker, SATB2, were upregulated. Furthermore, the expression of excitatory neuron marker, VGluT1, was upregulated. These results suggest that COSFs were differentiated to telencephalic fate and contained excitatory cortical neurons.

Immunohistochemistry for NeuN (mature neuronal marker), FoxG1, PAX6, Ki67, SATB2, TBR1, and CTIP2 was performed in COSFs and cCOs on day 70 (Fig. 2B, Supplementary Fig. S2G). The percentages of positive cells for each marker in COSFs/cCOs were as follows: NeuN, 31.5 ± 4.2%/31.0 ± 0.4%; FoxG1, 36.1 ± 3.8%/47.6 ± 1.4%; PAX6, 13.7 ± 3.2%/22.3 ± 6.1%; Ki67, 2.0 ± 0.7%/ 6.6 ± 2.2%; SATB2, 12.0 ± 2.4%/5.7 ± 3.0%; TBR1, 37.9 ± 5.5%/23.6 ± 2.3%; and CTIP2, 32.2 ± 4.0%/28.4 ± 1.9% (*n* = 3 individual COSFs/cCOs) (Fig. 2C). These results suggest that early-born cortical neurons (TBR1- or CTIP2-positive) comprised the major component (approximately 50%) of COSFs and that NS/PC components were still persistent. Late-born cortical neurons (SATB2-positive) were sufficiently differentiated at 70 days and were similar to cCOs (not statistically significant). Similar findings were observed for COSFs derived from 409B2 cells (Supplementary Fig. S2C, S2D). However, FoxG1 was not activated in the COSFs derived from 1201C1 cells, suggesting a failure to differentiate into the forebrain fate (Supplementary Fig. 4A, 4B). In the neural differentiation of hiPSCs, it is well known that there is cell-line-specific differentiation bias, which was also observed in our methods. The number of PAX6- and Ki67-positive cells in SiF sheets decreased dramatically from days 21 to 70 (Fig. 2D), indicating that NS/PCs in COSFs became less proliferative as its maturation progressed.

The expression of HOPX, which is the outer radial glia (RG) marker, was assessed to characterize these non-rosette-forming PAX6-positive cells in SiF sheets. A small amount of PAX6-positive cells coexpressed HOPX (9.5 ± 4.4%), suggesting that the SiF sheet of COSFs contained outer RG-like cells (Fig. 2E). In contrast, only a few glial fibrillary acidic protein (GFAP)-positive cells were observed in the SiF sheet (Fig. 2F), indicating that differentiation into astrocytes did not fully proceed. Outer RG-like HOPX-positive cells or GFAP-positive astrocytes were also observed as cCOs, but both of them are scarce (Supplementary Fig. 1G, 1H). As shown above, these data suggest that COSFs had differentiated into the cortical lineages with similar cell diversity to cCOs, while their cortical neuronal cell layers could expand larger into sheet-like configuration.

### Biased Localization of Progenitor Cell Population and Cortical Neuron in the Neuronal Layer of COSFs

**Figure 3.**
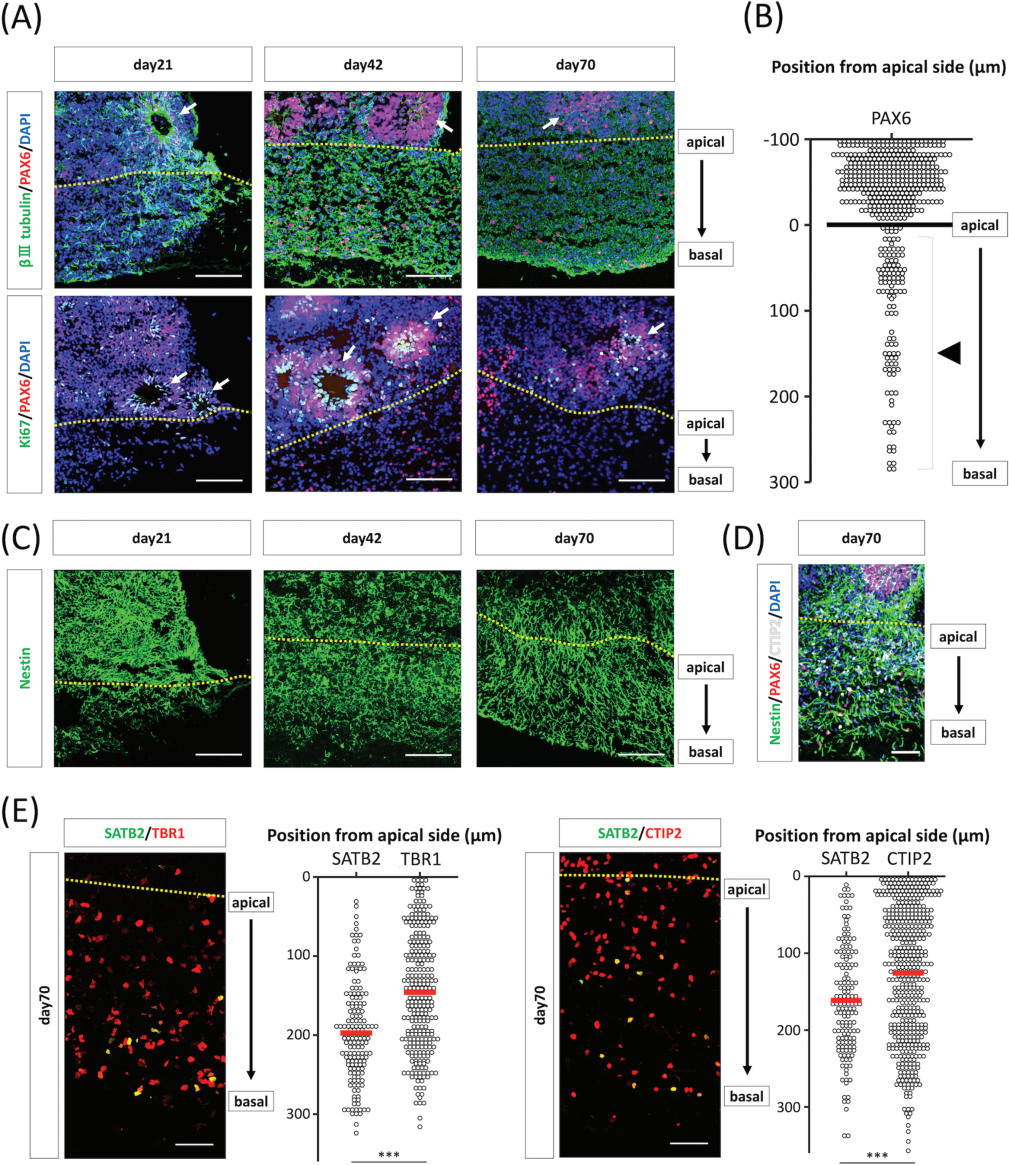
Localization of progenitor cell population and cortical neuron in the neuronal layer of cerebral organoids on silicate microfiber sheets (COSFs). (**A**) Immunohistochemistry for βIII tubulin and PAX6 on days 21, 42, and 70. On day 21, βIII tubulin-positive neurons expanded and reached the basal side of the SiF sheet. On day 42, apparent PAX6-positive neural rosettes were observed outside the silicate microfiber (SiF) sheets, which were filled with βⅢ tubulin-positive neurons. Immunohistochemistry for PAX6 and Ki67 revealed that neural rosette structures were not present inside the SiF sheet. The dotted line indicates the apical border of an SiF sheet. Arrows indicate neural rosettes. Scale bar = 100 µm. (**B**) Distribution of PAX6-positive nuclei in the COSFs on day 70. The distance of the SiF layer from the apical surface was measured. PAX6-positive cells are tightly clustered on and roughly inside the SiF sheet. *n* = 480 cells. The line indicates the apical border of an SiF sheet. Arrow head indicates the non-rosette forming PAX6-positive cells in the SiF sheet. (**C**) Immunohistochemistry for NESTIN showed that NESTIN-positive fibers expanded in the SiF sheets. The dotted line indicates the apical border of an SiF sheet. The dotted line indicates the apical border of an SiF sheet. Scale bar = 100 µm. (**D**) Immunohistochemistry for NESTIN, PAX6, and CTIP2 demonstrated that PAX6-positive neural rosettes were observed outside the SiF sheets, NESTIN-positive fibers expanded in the SiF sheets from neural rosettes, and CTIP2-positive neurons observed in the SiF sheets. Scale bar = 50 µm. (**E**) Distribution of SATB2-, TBR1-, CTIP2-positive nuclei in the SiF layer of the COSFs. The distance of the SiF layer from the apical surface (dotted line) was measured. Left: 158 cells for SATB2 and 273 cells for TBR1 from three individual COSFs. Right: *n* = 155 cells for SATB2 and *n* = 478 cells for CTIP2 from 3 individual COSFs. ***P < .001, Student’s *t* test.

PAX6- and Ki67- (proliferative cell marker) positive cells were analyzed to determine the spatial distribution of the stem/progenitor cell population in whole COSFs. We found that neural rosettes, in which neuroepithelial cells formed neural tube-like structures surrounding the ventricle-like inner lumen,^28^ were not present inside but formed outside the SiF sheet on days 21, 42, and 70 (Fig. 3A, 3B). On the apical side inside the SiF sheet, PAX6- and Ki67-positive cells were present without forming a rosette structure (Fig. 3A). NS/PCs marker, NESTIN, was expressed in the SiF sheet, and NESTIN-positive cell processes spread from apical to basal direction from days 21 to 70 (Fig. 3C, 3D). This was considered to be similar to the radial fibers observed in the developing cerebral cortex.

Immunohistochemistry of COSFs on day 70 was performed for the upper layer neuron (late-born) marker, SATB2, and lower layer neuron (early born) markers, TBR1 and CTIP2, to determine the spatial distribution of cortical neurons in the SiF sheets. SATB2-positive cells were distributed in a more basal side than the TBR1- or CTIP2-positive cells. We quantified the distance from the apical side of the SiF sheets and found that SATB2-positive cells were significantly located in the more basal side than TBR1- or CTIP2-positive cells (Fig. 3E). These data show that late-born neurons tended to be localized more in the basal side than early-born cortical neurons, suggesting that the cortical neuronal cell layers in the SiF sheets of COSFs are reminiscent of fetal brain-like inside-out structure. In addition, similar results were observed in 409B2 cells (Supplementary Fig. S2F--S2H).

### Neurites Outgrowth, Maturity, and Functionality of Cortical Neurons in COSFs

**Figure 4.**
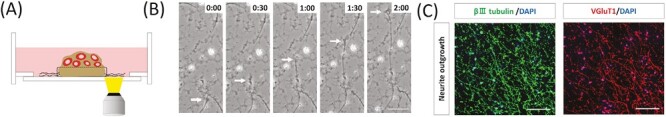
Observation of neurite outgrowth of the cortical neurons in cerebral organoids on silicate microfiber sheets (COSFs). (**A**) Schema of time-lapse imaging. The neurite outside of the sheet was observed. (**B**) Frames from live imaging showing neurite outgrowth. Time shown in hour:minute. Scale bar = 100 µm. (**C**) Immunohistochemistry for VGluT1 and βIII tubulin. Most neurites extending from COSFs were excitatory cortical neurons. Scale bar = 100 µm.

In the developing human cerebral cortex, juvenile cortical neurons polarize and extend the neurites to establish intracortical and subcortical projections,^29,30^ establishing the synaptic connection. We then assessed the neurite dynamics of COSFs. Time-lapse microscopy was performed in vitro to confirm the neurite extension activity of cortical neurons in COSFs. COSFs on day 70 were placed on matrix-coated glass bottom surface and cultured (Fig. 4A). During the fourth-day observation, neurites from the COSFs extended radially (Fig. 4B), with a growth speed of 44.5 ± 3.5 µm/h (*n* = 20 neurites). These neurons were confirmed to be VGluT1-positive excitatory neurons by immunohistochemistry for βIII tubulin and VGluT1 (Fig. 4C). These results show that the excitatory cortical neurons in COSFs had the potential to extend neurite actively.

**Figure 5.**
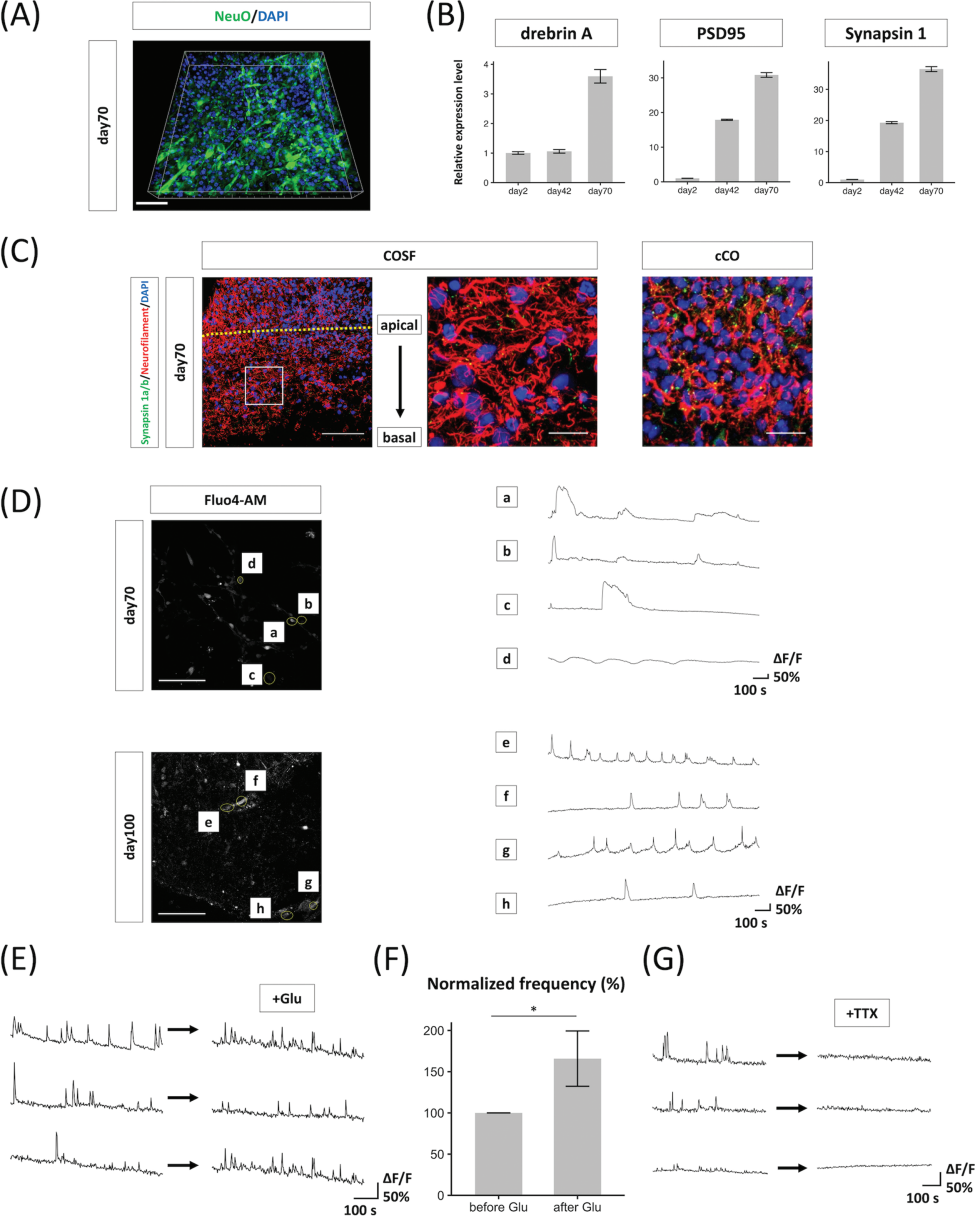
Functional maturity of cortical neurons in cerebral organoids on silicate microfiber sheets (COSFs). (**A**) Multiphoton microscopy imaging of NeuO revealed that the cortical neurons in COSFs extended each of the neurites in the parallel plane. Scale bar = 50 µm. (**B**) Quantitative reverse transcription-polymerase chain reaction analysis for the expression of the marker genes for pre- and post-synaptic marker in day 2, 42, and 70 COSFs. Relative expression levels normalized to the expression levels of day 2 are shown as fold change ± SEM, *n* = 3 technical replicates. (**C**) Immunohistochemistry for synapsin 1a/b and neurofilaments revealing that neurons in the SiF layers and cerebral organoids (COs) had synaptic vesicles. Scale bar = left 200 µm, right 20 µm. (**D**) Analysis of calcium surges in individual neurons in the silicate microfiber (SiF) layer on days 70 and 100. Scale bar = 100 µm. (**E**) Analysis of calcium surges before and after the application of glutamate (Glu) on day 101. (**F**) Quantitative evaluation of spike frequencies before and after the application of Glu normalized to the frequency before application. *n* = 12 cells from three individual COSFs. Results are presented as mean ± SEM. *P < 0.05, Student’s *t*-test. (**G**) Analysis of calcium surges before and after the application of tetrodotoxin (TTX) on days 100 and 102.

Cortical neurons in COSFs on day 70 were fluorescently visualized through NeuO staining to visualize the neural networks 3-dimensionally in SiF sheets. The 3D reconstruction of multiphoton microscopy-acquired images showed that the neurites of the neurons in the SiF sheets of COSFs were extended horizontally and tangled with each other (Fig. 5A). Quantitative RT-PCR analysis of COSFs on days 2, 42, and 70 revealed that the expressions of the pre- and postsynaptic markers (SYNAPSIN1, DREBRIN A, and PSD95, respectively) were all increased (Fig. 5B). The immunohistochemical analysis of the SiF sheets of COSFs and cortical layers of COs confirmed the expression of Synapsin1, which is a marker of synaptic vesicles (Fig. 5C), although they are infrequent. Given the insufficient localization and expression of pre-and postsynaptic proteins, we concluded that the cortical neurons in the SiF sheets of COSFs developed some synaptic connections, but they were considered to be still immature.

Finally, multiphoton calcium imaging was performed to assess the functionality of the cortical neuron in the SiF sheets of COSFs. Ca^2+^ indicator dyes were used to confirm the neuronal activity of cortical neurons in COSFs. We successfully detected uneven spontaneous calcium bursts and waves in COSFs on day 70 and sharp unitary or multipeak spikes in COSFs on day 100 (Fig. 5D). The frequency of these spontaneous firings increased with glutamate treatment, indicating glutamatergic receptor activity (Fig. 5E, 5F). Furthermore, tetrodotoxin was applied to block action potentials and observed that calcium surges were suppressed, suggesting that calcium fluctuations are dependent on neuronal activity (Fig. 5G).

However, there was little synchronized activity in these cortical neurons in COSFs (mean correlation coefficient value: 0.28 ± 0.055 [day 70] and −0.011 ± 0.083 [day 100], *n* = 7 cells simultaneously recorded). These results show that each neuron in COSFs had differentiated into electrically functional cortical neurons, although their synaptic connectivity was still immature. These results were consistent with the results of immunostaining.

## Discussion

In this study, we developed a novel method to enhance the growth of the cortical neuronal layers of COs using SiF scaffolds. Similar to the conventional methods,^3^ pluripotent stem cells differentiated into neuroepithelial cells and autonomously organized the tissue-like structures with the SiF scaffolds. In contrast to the conventional methods, neuronal cell layers were formed inside the scaffolds and thicker than that of conventional organoids. The cortical neuron in the scaffolds exhibited the electrical activities and vigorous capacity of neurites extension, suggesting that they were functionally differentiated cortical neurons.

Our COSFs were able to sustain 70 days of incubation maintaining their growth and viability without collapse regardless of their large size. The growth and maturation of COs require a longer time because in vitro corticogenesis requires a time roughly equivalent to that of human embryogenesis in utero.^31^ Long-term culture in vitro is still challenging because the microenvironment, including the nutrition and oxygenation supply of the core of COs, deteriorates with growth.^32^ Elaborative research has been conducted to develop methods to supply oxygen and nutrients through the spinning bioreactor,^33^ shaking culture,^16^ high-oxygen conditions,^2^ organotypic slice culture,^18,34^ and vascularization.^35^

The supportive effects of SiF scaffolds on COs were largely attributed to their fine random fibers, highly porous structure, and robust framework. Microfiber scaffolds are known to elicit differentiation, proliferation, tissue formation, and in vivo metabolism in the 3D culture of neural stem cells and various tumors,^36-40^ and similar findings were obtained in COSFs. In addition, we speculated that other mechanisms may have helped the reproduction of unique cell dynamics, which was observed during corticogenesis. One possibility is the sieve-like function of SiF. The flow pore size of scaffolds is estimated to be 7-8 μm,^26^ which enables the extending radial processes or migrating cells in locomotion or translocation to pass; however, tightly adhered epithelial cells, which form neural rosettes, could not enter the scaffold layer. This speculation is consistent with our observation that neuroepithelium-like rosettes were located on the surfaces of scaffolds, while radial glial-like processes, progenitor cells, or cortical neurons abundantly spread into the scaffolds. This sieve-like function might separate neuroepithelial cells from differentiated progenies and contribute to the sound cell-migration and organization of the cortical neuronal layer in vitro. Another possibility is the physical support from SiF. Meanwhile, the fate specification of pluripotent stem cells into the cerebral cortex excluding non-neural lineage leads to adverse effects on the development of mesenchymal structures (eg, meninges) in COs.^41^ SiF scaffolds might fill in for them and protect against physical injury during long-term spinner culture.

The cortical neuronal layer of COs is still underdeveloped specifically its thickness, which is not yet comparable to that of the human brain.^27^ We successfully developed methods to generate COs with a thick cortical neuronal layer, although their architecture was still rudimental and architectonic compartments (ie, subventricular zone, intermediate zone, subplate, cortical plate, and marginal zone) were not clearly distinguishable, similar to the fetal brain^27,42^; however, the distribution of late- and early-born cortical neurons were biased according to the ontogenic manner. To model the architecture of the fetal human brain precisely, additional cues from various extracellular matrices might be required, because they had pivotal function in the neuronal migration and lamination in the rodent developing brain.^43^ Contrary to the simple bioengineering approach of piling scaffold-embedded cortical neurons up to construct cerebral cortex-like tissue,^44^ our approach is based on the self-organization process, which was the basic principle of embryogenesis. Thus, our cortical model may be useful for the research on human cerebral cortex development and malformation, such as lissencephaly and cortical dysplasia.

In addition, our COSFs had sheet-like configuration, which is a unique feature that cannot be achieved by conventional organoid technology. Sheet-like modules of the human cerebral cortex could be applicable for the integration of organ-on-a-chip devices with microfluidic devices or electrophysiological equipment, which may be a recapitulation of the pharmacological effects on the living brain. Furthermore, the integration of this artificial cerebral cortex-like tissue with the human living brain may potentially be applied in medical practice.^45^ While human brain-derived resources are very difficult to obtain, our technology may be used to obtain human-derived living tissue to assemble these systems without ethical issues and enormous expense. COSFs may be integrated into the adjacent host cerebral cortex given their robust ability to extend neurites, as demonstrated previously using rodent embryonic cortical tissue^46,47^ or human cCO.^48,49^ Our COSFs were larger than cCOs and had a sheet-like configuration, which would be suitable for human cerebral cortex regeneration.

However, we recognized some limitations in our methods. Similar to other neural differentiation methods, the efficiency of cortical differentiation is greatly influenced by the inherent propensity of the iPSC lines. Therefore, we prioritized FoxG1 as a differentiation marker for the forebrain. This marker is important, because there is a significant variation between iPSC lines during neural differentiation.^50^ We also recognized another limitation concerning the incomplete maturation of synaptic connections, similar to other in vitro culture systems.^51^ To tackle this concern, we are considering the maturation of synapses by incorporating other cell types such as astrocytes and oligodendrocytes, found in the adult brain, and by integrating them with bioreactor devices that mimic the circulatory system, which recapitulates the environment such as transplanted CO models with mature synaptic connections.^49^

In summary, SiF scaffolds are promising resources for the enhanced growth of the cortical neuronal layers of COs. In vitro scaffold-supported corticogenesis is a novel concept, which may be applicable for the fabrication of innovative bio-products or regenerative medicine.

## Conclusion

We established methods for generating sheet-like and thick cortical neuronal layers of human iPSC-derived COs. Our methods would be a promising approach in the research on human corticogenesis, pharmaceutical applications, and regenerative medicine for diseases in the cerebral cortex.

## Supplementary Material

szad066_suppl_Supplementary_Tables_1-2_Figures_1-3

## Data Availability

The datasets generated and/or analyzed during the current study are available from the corresponding author on reasonable request.
