# Supplementary material for: Silicate Microfiber Scaffolds Support the Formation and Expansion of the Cortical Neuronal Layer of Cerebral Organoids With a Sheet-Like Configuration
Source: Stem Cells Transl Med. 2023 Oct 16;12(12):825–37. doi: 10.1093/stcltm/szad066 (PMC10726412; doi:10.1093/stcltm/szad066)
Supplement: szad066_suppl_Supplementary_Tables_1-2_Figures_1-3 [file szad066_suppl_supplementary_tables_1-2_figures_1-3.pdf]

Supplementary Table 1

Primers for quantitative PCR

**Gene**	**Forward**	**Reverse**
CTIP2	*5'-GAGTACTGCGGCAAGGTGTT-3'*	*5'-TAGTTGCACAGCTCGCACTT-3'*
drebrinA	*5'-TTCATAAAGGCATCGGACAGTGG-3'*	*5'-ATGGGAGGGAGGAAGAGAGGTTTGG-3'*
FoxG1	*5'-GCCACAATCTGTCCCTCAACA-3'*	*5'-CGGGTCCAGCATCCAGTAG-3'*
GAPDH	*5'-CGCTCTCTGCTCCTCCTGTT-3'*	*5'-CCATGGTGTCTGAGCGATGT-3'*
Oct4	*5'-TGTACTCCTCGGTCCCTTTC-3'*	*5'-TCCAGGTTTTCTTTCCCTAGC-3'*
PAX6	*5'-GTGTCTACCAACCAATTCCACAAC-3'*	*5'-CCCAACATGGAGCCAGATG-3'*
PSD95	*5'-TCGGTGACGACCCATCCAT-3'*	*5'-GCACGTCCACTTCATTTACAAAC-3'*
SATB2	*5'-CCTCCTCCGACTGAAGACAG-3'*	*5'-TGGTCTGGGTACAGGCCTAC-3'*
SOX1	*5'-GCAAGATGGCCCAGGAGAA-3'*	*5'-CCTCGGACATGACCTTCCA-3'*
synapsin1	*5'-GACGGAAGGGATCACATCAT-3'*	*5'-CTGGTGGTCACCAATGAGC-3'*
TBR1	*5'-ATGGGCAGATGGTGGTTTTA-3'*	*5'-GACGGCGATGAACTGAGTCT-3'*
VGluT1	*5'-GAAACTCATGAACCCCCTCA-3'*	*5'-GGGAGATGAGCAGCAGGTAG-3'*

Supplementary Table 2

The antibodies used in this study

Antibody	Source	Company	Identifier	Dilution
**Primary antibodies**				
Cleaved Caspase-3	rabbit polyclonal	cell signaling	#9661	1:400
CTIP2	rabbit polyclonal	abcam	ab28448	1:500
CTIP2	rat monoclonal	abcam	ab18465	1:500
FoxG1	rabbit polyclonal	abcam	ab18259	1:1000
GFAP	rabbit polyclonal	abcam	ab7260	1:500
HOPX	mouse monoclonal	Santa Cruz	sc-398703	1:100
Ki67	mouse monoclonal	BD biosciences	#550609	1:50
Nestin	mouse monoclonal	abcam	ab22035	1:1000
Neurofilament L	rabitt monoclonal	cell signaling	#2837	1:100
NeuN	rabbit monoclonal	cell signaling	#24307	1:300
PAX6	rabbit polyclonal	BioLegend	#901301	1:500
SATB2	mouse monoclonal	Santa Cruz	sc-81376	1:100
Synapsin 1a/b	mouse monoclonal	Santa Cruz	Sc-376623	1:100
TBR1	rabbit polyclonal	abcam	ab31940	1:500
TUJ1	Mouse monoclonal	BioLegend	#801201	1:1000
VGluT1	rabbit polyclonal	Synaptic Systems	#135302	1:500
**Secondary antibodies**				
Alexa Fluor 488		abcam	ab150113	1:500
Alexa Fluor 568		abcam	ab175471	1:500
Alexa Fluor 647		abcam	ab150159	1:500
DAPI		nacalai tesque	28718-90-3	1:1000

Supplementary Figure 1

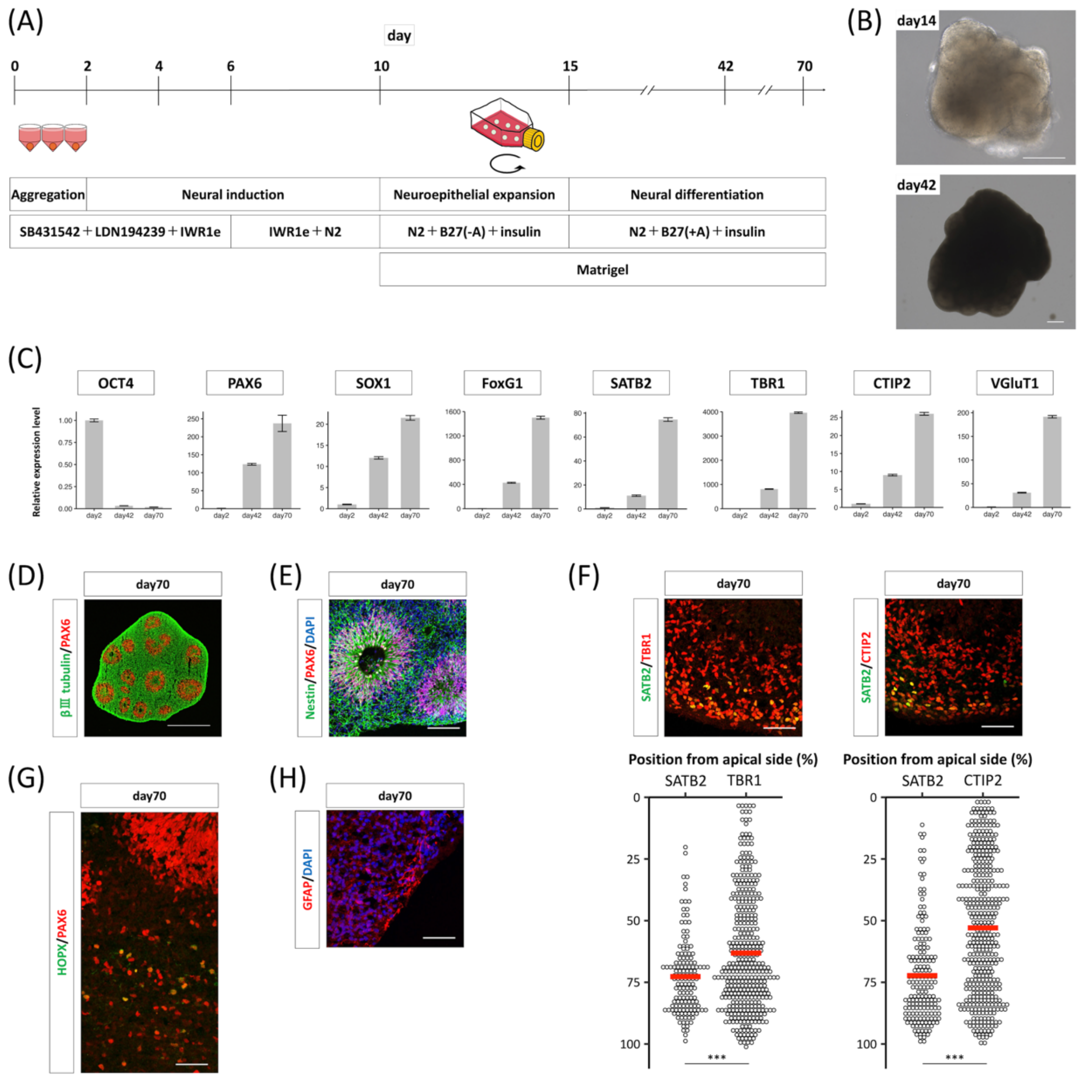

Supplementary figure 1. Generation and characterization of cerebral organoids (COs) without silicate microfiber (SiF) sheets (conventional CO, cCO) using 201B7 cells.

A. Procedure for the derivation of COs without SiF scaffolds.

B. Phase-contrast images of cCO on days 14 and 42. Scale bar, 300 μm.

C. Quantitative reverse transcription-polymerase chain reaction analysis for the expression of the marker genes for neural lineage and undifferentiated stem cells in cCOs on days 2, 42, and 70. Relative expression levels normalized to the expression levels in cCOs on day 2 are shown as fold change ± standard error of the mean, n = 3 technical replicates.

D. Immunohistochemistry for βIII tubulin and PAX6 on day 70. PAX6-positive neural rosettes were observed, forming βIII tubulin-positive neurons around the rosettes. Scale bar, 500 μm.

E. Immunohistochemistry for NESTIN showed that NESTIN-positive fibers expanded from the PAX6-positive neural rosettes. Scale bar, 100 μm.

F. Distribution of SATB2-, TBR1-, CTIP2-positive nuclei within the cortical plate in cCOs. For relative positions, the apical and basal boundaries of the cortical plate were defined as 0 to 100. Left: 145 cells for SATB2 and 386 cells

for TBR1 from eight individual cCOs. Right: n = 177 cells for SATB2 and n = 411 cells for CTIP2 from eight individual cCOs. *** $P < 0.001$, Student's t test. Scale bar, 50 μm.

G. Immunohistochemistry for PAX6 and HOPX revealed the presence of outer radial glial-like cells in cCOs. Scale bar, 50 μm.

H. Immunohistochemistry for glial fibrillary acidic protein and 6-diamidino-2-phenylindole dihydrochloride revealed that there were a few astrocytes in cCOs at day 70. Scale bar, 50 μm.

Supplementary Figure 2

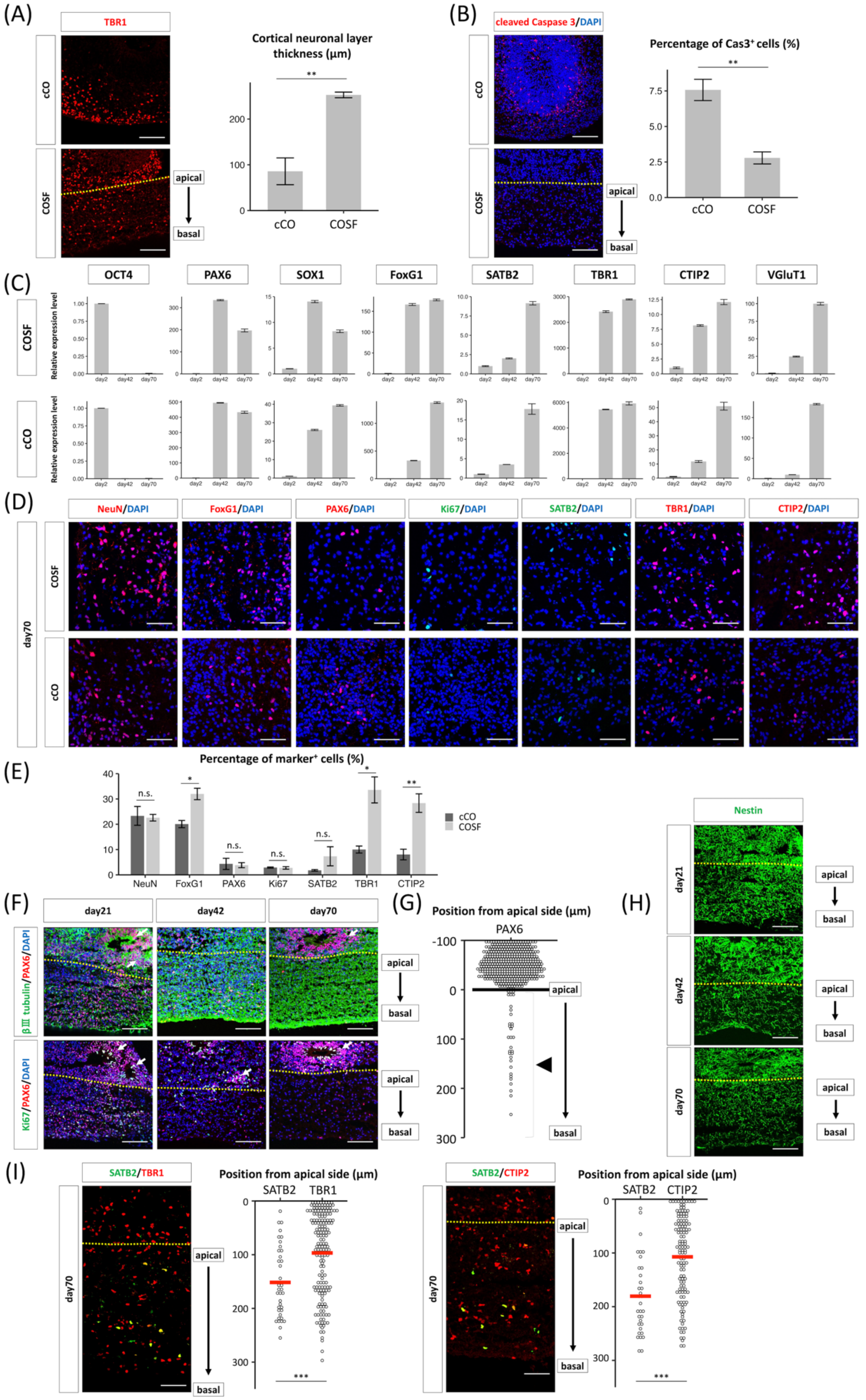

Supplementary figure 2. Features of cerebral organoids on silicate microfiber sheets (COSFs) using 409B2 induced pluripotent stem cells.

A. Comparison of TBR1-positive cortical neuronal layer thickness among COs and COSFs. In 70 days, the cortical thickness was 252.8 ± 6.1 μm (COSFs) and 85.8 ± 29.3 μm (cCOs). n = 3 individual COs and 3 points from three individual COSFs. Results are presented as mean ± standard error of the mean. $^{**}P < 0.01$, student's t test. Scale bar, 100 μm.

B. Comparison of the viability rates between cCOs and COSFs. The dotted line indicates the apical border of an SiF sheet. n = 3 individual cCOs and COSFs values. Results are presented as mean ± standard error of the mean. $^{**}P < 0.01$, Student's t test. Scale bar, 100 μm.

C. Quantitative reverse transcription-polymerase chain reaction analysis for the expression of the marker genes for neural lineage and undifferentiated stem cells in COSFs and cCOs on day 2, 42, and 70 COSFs. Relative expression levels normalized to the expression levels in COSFs on day 2 are shown as fold change ± standard error of the mean, n = 3 technical replicates.

D, E. Immunohistochemistry and quantification of NeuN-, FoxG1-, PAX6-, Ki67-, SATB2-, TBR1-, and CTIP2-positive cells in the SiF layer of COSFs and cCOs at day 70. Scale bar, 50 μm. n = 3 individual COSFs and cCOs on day 70. Results are presented as the mean ± standard error of the mean. $^{*}P < 0.05$, $^{**}P < 0.01$, Student's t test.

F. Immunohistochemistry for βIII tubulin and PAX6 on days 21, 42, and 70. On day 21, βIII tubulin-positive neurons expanded and reached the basal side of the SiF sheet. On day 42, apparent PAX6-positive neural rosettes were observed outside the silicate microfiber (SiF) sheets, which were filled with βIII tubulin-positive neurons. Immunohistochemistry for PAX6 and Ki67 revealed that neural rosette structures were not present inside the SiF sheet. Dotted line indicates the apical border of an SiF sheet. Arrows indicate neural rosettes. Scale bar, 100 μm.

G. Distribution of PAX6-positive nuclei in the COSFs on day 70. The distance of the SiF layer from the apical surface was measured. PAX6-positive cells are tightly clustered on roughly inside the SiF sheet. n = 437 cells. The line indicates the apical border of an SiF sheet. Arrowhead indicates the non-rosette forming PAX6 positive cells in the SiF sheet.

H. Immunohistochemistry for NESTIN showed that NESTIN-positive fibers expanded in the SiF sheets. Dotted line indicates the apical border of a SiF sheet. Scale bar, 100 μm.

I. Distribution of SATB2-, TBR1-, CTIP2-positive nuclei in the SiF layer of the COSFs. The distance of the SiF layer from the apical surface (dotted line) was measured. Left: 39 cells for SATB2 and 171 cells for TBR1 from three individual COSFs. Right: n = 33 cells for SATB2 and n = 125 cells for CTIP2 from three individual COSFs. Student's t test. Scale bar, 50 μm.

Supplementary Figure 3

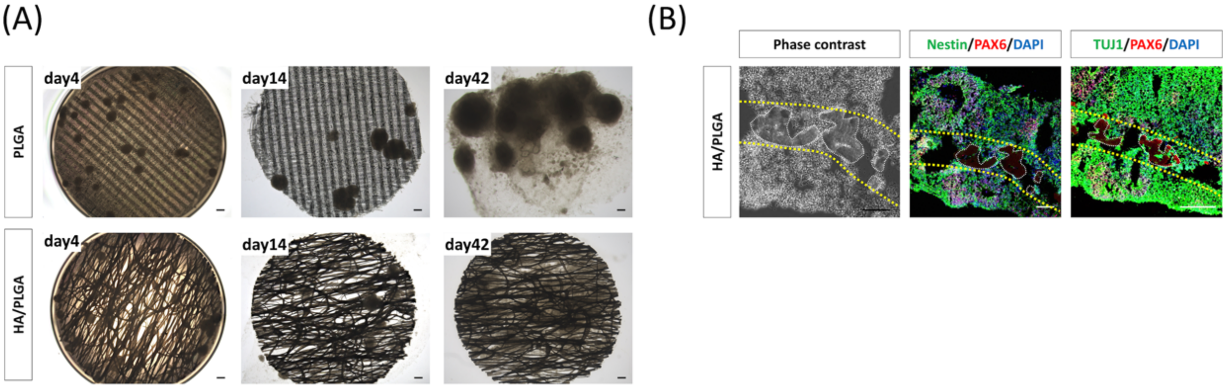

Supplementary figure 3. Specific interaction of various scaffolds with cerebral organoids.

A. Microscopic images of scaffold culture using poly(lactide-co-glycolide) copolymer (PLGA) mesh and hydroxyapatite (HA)/PLGA composites on day 4, 14, and 42. PLGA mesh dissolves and loses its original shape in long-term incubation. Scale bar, 300 µm.

B. Phase contrast image and Immunohistochemistry for NESTIN, βIII tubulin, and PAX6 on day 42. Adhering to the fibers, NESTIN-positive fibers expanded from the PAX6-positive neural rosettes and βIII tubulin-positive neurons formed around the rosettes. Neural rosettes were present mainly on the surface of the scaffolds, and some plunged into the spaces between thick HA/PLGA fibers, suggesting that cortical cell layers were not formed in the scaffolds. Scale bar, 100µm.

Supplementary Figure 4

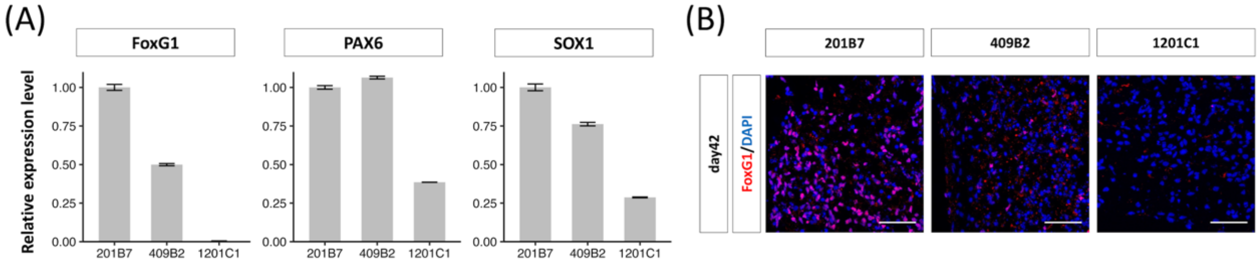

Supplementary figure 4. Variation of the propensity in differentiation to forebrain fate among induced pluripotent stem cells lines.

A. Quantitative reverse transcription-polymerase chain reaction analysis for the expression of the marker genes for forebrain and neural stem cells in COSFs on day 42 COSFs. Relative expression levels normalized to the expression levels in COSFs derived from 201B7 line are shown as fold change ± standard error of the mean, n = 3 technical replicates.

B. Immunohistochemistry for FoxG1 revealed that COSFs derived from 1201C1 were not induced to the forebrain. Scale bar, 100 µm.
